# Editorial: Evaluative complexities in forensic psychology: methodological and ethical considerations

**DOI:** 10.3389/fpsyg.2026.1932846

**Published:** 2026-08-26

**Authors:** Guido Travaini, Monia Vagni, Martino Ongis

**Affiliations:** 1 Vita-Salute San Raffaele University, Milan, Italy; 2 Department of Philosophy, Social Sciences, Humanities and Education, University of Perugia, Perugia, Italy; 3 Franklin Switzerland, Lugano, Switzerland

**Keywords:** expert evidence, forensic—psychiatric practice, forensic psychology, med-legal, testimonial accuracy

## Introduction

Forensic psychology and forensic psychiatry stand at a difficult threshold: they translate mental states, behavior, memory, risk, and vulnerability into assessments that can shape liberty, protection, clinical care, and public trust in justice. This Research Topic was built around a premise that is at once methodological and ethical: evaluative complexity is not a peripheral inconvenience but the object of the field. Uncertainty, disagreement, contextual vulnerability, and measurement limits must be identified, documented, and managed rather than obscured.

The 12 contributions collected here address the three aims of the Topic—offender treatment and correctional evaluation, variability and objectivity in medico-legal and psycho-criminological assessment, and testimonial capacity—while widening them to courtroom evidence, correctional mental health, biometric decision support, abusive group dynamics, and psychotherapeutic confidentiality. Across these areas, legal categories are indispensable but never self-validating. They must be translated into psychological questions, examined with appropriate methods, and returned to legal decision-makers with their scope and limits intact.

## Contributions to the Research Topic

At the epistemological core of the Research Topic, Verde and Gritti reconceptualize forensic psychological assessment as a multilevel abductive process in which idiographic case formulation must be anchored to nomothetic data and protected from confirmation bias, first-impression effects, and self-presentation distortions. Their contribution is complemented by Duggan and Sarkar, who examine the courtroom difficulty of applying group evidence to an individual person. Together, these articles argue that expert opinion gains authority not by suppressing uncertainty, but by making its assumptions, inferential steps, and limits explicit. This is especially important in adversarial settings, where confidence can be mistaken for validity and where a transparent account of uncertainty may be more helpful to justice than an overprecise conclusion.

A second cluster concerns testimony, memory, and evidentiary reasoning. Pacchioni et al. show that Italian Supreme Court decisions concerning testimony after EMDR therapy do not support categorical assumptions that the treatment is intrinsically suggestive; rather, judicial scrutiny turns on case-specific evidence, transparent documentation, and the separation of therapeutic and investigative roles. Gudjonsson et al. refine this problem experimentally by distinguishing omission- and commission-oriented memory distrust and their different relationships with immediate and delayed suggestibility. Denne et al. add a sobering view from child sexual abuse exonerations: features often treated as suspicious or corroborative may appear in both true and false allegations, making careful interviewing, corroboration, and case-specific analysis indispensable.

The same concern for human decision-making under uncertainty extends to technologies and psychophysiological tools. Naser et al. demonstrate that the interface of an automatic face recognition system can change false-alarm risk, with simultaneous presentation reducing false alarms in target-absent conditions. Orthey et al. show that the Concealed Information Test cannot simply extrapolate from young, healthy samples to older examinees: skin conductance, heart rate, and respiration are differently affected by age, requiring cautious interpretation and further validation.

A third cluster turns to correctional and forensic clinical settings. Torales et al. report a substantial screening-positive burden of ADHD symptoms among incarcerated people in Paraguay, particularly among women, and identify associations with suicidality and emotional dysregulation while appropriately distinguishing screening from diagnosis. Fojcik et al. examine subjective mood among forensic psychiatric inpatients, finding generally low depressive symptoms but greater severity with longer hospitalization. Both studies show why forensic care must include systematic monitoring without reducing individuals to institutional status or single diagnostic labels.

Developmental and contextual complexity is foregrounded by Liu et al., who compare juvenile offenders and juvenile criminals in China. Their findings challenge any easy assumption that legal categories map onto lower psychological risk and support differentiated, empirically monitored interventions. In parallel, Tinelli et al. examine Italian psychologists' awareness of group psychological abuse, revealing limited formal preparation, reliance on non-academic sources, and a need for clearer terminology, assessment tools, and professional training. These papers remind us that forensic categories often originate in law, institutions, or public discourse, but their use in evaluation requires independent psychological validation. The evaluator's task is therefore translational: to respect the legal question while resisting the temptation to make clinical science say more, or less, than the evidence permits.

Finally, John et al. address one of the oldest tensions in clinical-forensic practice: confidentiality and mandatory reporting. Across jurisdictions, the protection of vulnerable people may require disclosure, yet overbroad or poorly explained duties can compromise therapeutic trust and access to care. The article frames law not as a background constraint but as an active determinant of clinical decision-making, risk management, and ethical accountability.

## Cross-cutting implications

Across these diverse contributions, four directions for the field emerge. First, forensic conclusions should disclose their inferential architecture: the data considered, hypotheses rejected, degree of uncertainty, and reasons why a conclusion is warranted. Second, instruments and procedures require validation in the populations and settings in which they are used, including older adults, incarcerated people, juveniles, witnesses exposed to therapy, and professionals interpreting technological outputs. Third, testimony and identification evidence demand safeguards against both naive skepticism and naive credulity; neither the presence nor the absence of familiar case features should substitute for disciplined analysis. Fourth, ethics is not an appendix to methodology. Documentation, role separation, proportionality, cultural and jurisdictional sensitivity, and professional training are conditions of valid evaluation. A further implication is that disagreement among experts should not be dismissed as professional noise. When systematically examined, disagreement can reveal hidden assumptions, poorly specified referral questions, inadequate instruments, or contextual factors that require further investigation.

## Conclusion

The central message of this Research Topic is that forensic evaluation should not aspire to a rhetoric of perfect certainty. Its task is more demanding: to produce warranted, transparent, and revisable conclusions in settings where errors may affect liberty, dignity, treatment, family life, and public safety. The contributions collected here show a field moving toward methodological humility without abandoning scientific ambition. They invite future research that integrates empirical validation, legal literacy, clinical expertise, and ethical reflexivity. Such research should prioritize ecologically valid samples, transparent analytic choices, training for professionals who handle high-stakes evidence, and procedures that make case formulation auditable. In that integration lies the credibility of forensic psychology and psychiatry as sciences of evaluation and as practices of justice.

